# An Unusual Variant of a Common Palatal Salivary Gland Tumor: Case Report of a Pleomorphic Adenoma with Significant Lipomatous Metaplasia

**DOI:** 10.1155/2018/2052347

**Published:** 2018-12-25

**Authors:** Sonal S. Shah, Tamer Zayed Moustafa

**Affiliations:** New York University College of Dentistry, 345 E 24th St, Room 837, NY, New York 10010, USA

## Abstract

**Introduction:**

Salivary gland tumors are relatively common in the junction of the hard and soft palate area of the oral cavity. Pleomorphic adenoma is considered the most common benign salivary gland tumor in this location. Some of the rarer subtypes of this tumor may have a misleading clinical presentation. Recognition of these variants is important since long-standing pleomorphic adenomas have the potential to become malignant.

**Case Presentation:**

A healthy 24-year-old male was referred for a painless, large, slowly growing, exophytic swelling of the right hard and soft palate. Interestingly, the lesion was yellowish in color and soft to palpation, suggestive of an innocuous lipoma or cystic lesion. An incisional biopsy was performed and the diagnosis was consistent with pleomorphic adenoma with a significant adipose tissue component. The patient was referred to an oral surgeon and underwent a complete surgical excision. Upon two-year follow-up, the patient is doing well with no recurrences.

**Conclusion:**

This case highlights a rare microscopic variant of pleomorphic adenoma with altered clinical presentation that led to an erroneous clinical diagnosis. The importance of taking a biopsy for definitive diagnosis and appropriate management is reinforced.

## 1. Introduction

Pleomorphic adenoma (PA) is by far the most common benign salivary gland neoplasm, accounting for 70-80% of total salivary gland tumors [1]. This tumor is characterized by neoplastic proliferation of glandular cells along with myoepithelial components. The term “pleomorphic” is used to describe the histology and histogenesis of the tumor cells and derives from the Greek words for “more” and “form”. The vast majority of cases involve the parotid gland, mainly the tail or lower pole, according to the World Health Organization. Intraoral pleomorphic adenomas occur in the minor salivary glands (often in the palate), submandibular glands, and rarely the sublingual glands [1].

Pleomorphic adenoma is categorized microscopically into 3 broad types: classic, cellular, and myxoid [2]. However, fat-containing benign tumors of the salivary glands, also known as lipomatous pleomorphic adenomas (LPA), are fairly rare. The histomorphological spectrum varies from minor scattered fat cells in the tumor up to pure lipomatous mesenchymal lesions that are difficult to distinguish from adipocytic neoplasms [2]. The World Health Organization has defined LPA as pleomorphic adenomas with a 90% or higher adipose component in the stroma [2].

In the following report, we present an uncommon case of a pleomorphic adenoma of the palate that clinically presented in an unusual manner. Due to the significant lipomatous component, the lesion had a yellowish color and felt soft to palpation, leading to an erroneous clinical diagnosis of lipoma. We stress the importance of biopsy, especially utilization of the punch biopsy technique, for definitive diagnosis and the need for oral health care providers to be aware of variations of the typical clinical appearance of pleomorphic adenomas.

## 2. Case Presentation

**Figure 1:**
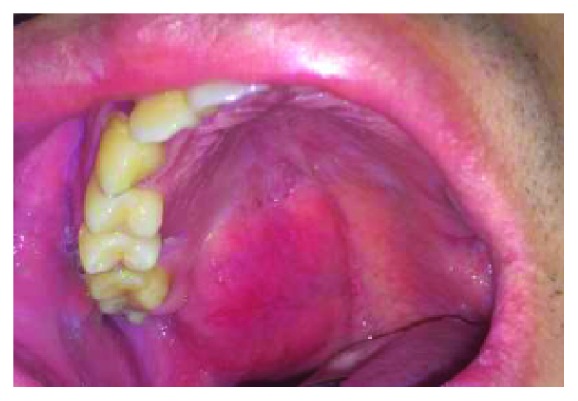
Lesion at the initial visit. Note the diffuse nature of the lesion, the yellowish hue, and surface vasculature.

**Figure 2:**
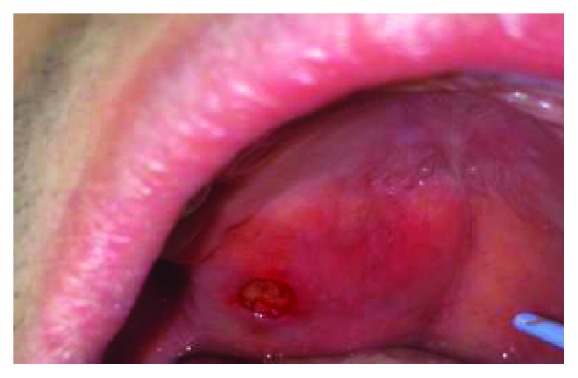
Immediately after the punch biopsy was performed, yellowish fat-like material is seen.

**Figure 3:**
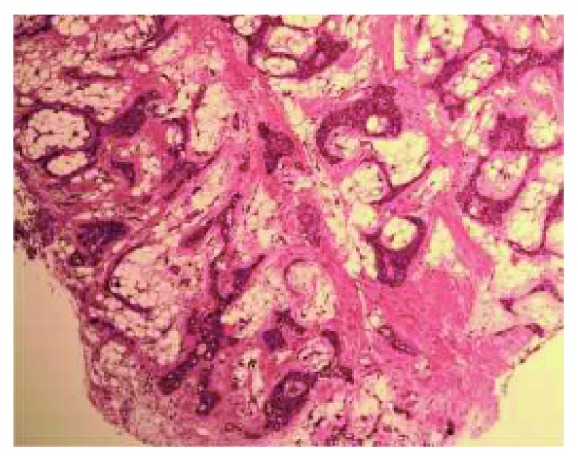
Low-power photomicrograph showing cellular areas with abundant intervening adipose tissue (4x original magnification, hematoxylin and eosin stain).

**Figure 4:**
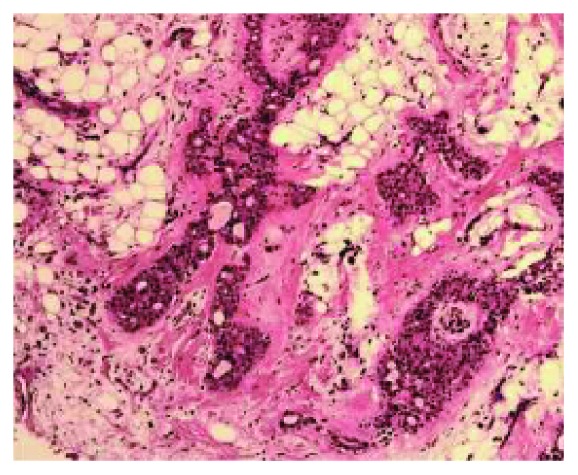
Ductal structures, hyalinization and adipose tissue can be seen in this medium-power view. (10x original magnification, hematoxylin and eosin stain).

**Figure 5:**
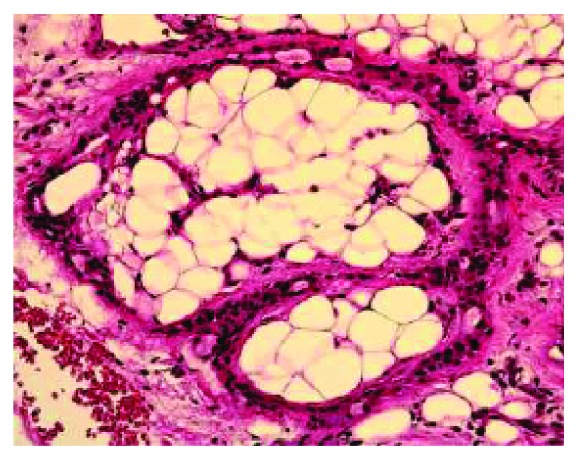
High-power photomicrograph of adipose tissue stroma surrounded by ducts (20x original magnification, hematoxylin and eosin stain).

**Figure 6:**
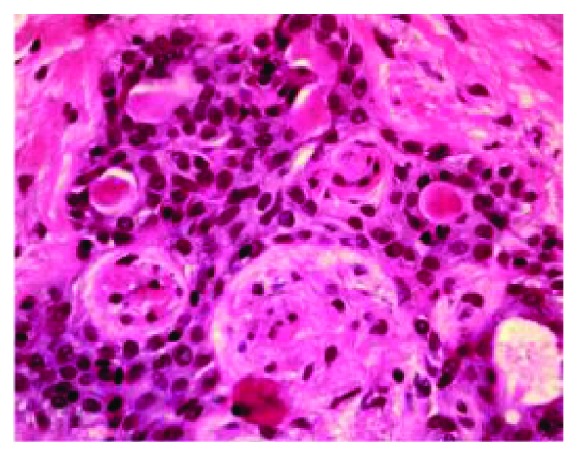
High-power photomicrograph showing double-layered ducts and plasmacytoid cells, characteristic features of the pleomorphic adenoma. (40x original magnification, hematoxylin and eosin stain).

A 24-year-old male of Asian descent reported to the oral medicine clinic at NYU College of Dentistry. His chief concern was a painless, slowly growing mass on his hard palate that he noticed a few weeks ago. The patient denied any significant medical issues or medications. He also denied any significant family history or any similar lesions in any of his immediate family members. The extraoral examination was within normal limits. Intraoral examination revealed a large exophytic mass of the right hard palate extending to the soft palate, yellowish in color, and soft to palpation (Figure 1). The lesion measured approximately 5 × 4 cm and was oval-shaped. It was compressible and had a smooth surface with numerous small blood vessels. However, the mass did not blanch or feel pulsatile upon palpation, ruling out a vascular tumor. The lesion felt fixed with well-defined margins. The working or clinical diagnosis was lipoma. The likely differential diagnosis included lipoma, a cystic lesion or other soft tissue tumor, and pleomorphic adenoma. A 5 mm incisional punch biopsy was performed in the center of the mass (Figure 2). On microscopic examination, a benign salivary gland tumor consisting of pools of plasmacytoid cells and numerous double-layered ducts was seen. The stroma was composed of significant areas (approximately 50%) of adipose tissue, along with several foci of hyalinization (Figures 3–6). The final diagnosis rendered was pleomorphic adenoma with significant adipose tissue component. The patient was then referred to oral surgery for complete surgical excision. A CBCT was performed to further delineate the lesion and confirm its benign behavior. No other investigations or diagnostic tests were performed. Surgical excision was completed and the pathology findings were consistent with the incisional biopsy results of pleomorphic adenoma with significant adipose tissue component (also approximately 50%). Upon 2-year follow-up, the patient is doing well and has no recurrences.

## 3. Discussion

Pleomorphic adenoma (PA) is the most common benign salivary gland tumor with the parotid gland being the most common site. Histopathologically, it is a benign neoplasm with myoepithelial and epithelial components arranged in diverse morphological patterns. The epithelial cells typically form duct-like structures or pools of plasmacytoid myoepithelial cells. The stromal element demonstrates varying degrees of myxoid, hyaline, cartilaginous, or osseous differentiation [3]. A lipomatous component may also be seen but prominent lipomatous differentiation is a rare variation of pleomorphic adenoma [4].

The term “lipomatous” pleomorphic adenoma was first used by Seifert et al. in 1999. According to Seifert et al., a lipomatous pleomorphic adenoma (LPA) consists of a stroma that is more than 90% lipomatous [5]. In another major classification system, the fat-containing salivary gland tumors can be categorized into 2 main groups based on their histopathological composition: monophasic true adipocytic neoplasms (lipoma and its variants) and hybrid lipoepithelial lesions composed of epithelial variants mixed with a variable fatty component [2]. Another accepted classification divides fat-containing tumors and tumor-like lesions of salivary glands into 4 categories: 1—fat containing epithelial/myopithelial tumors such as pleomorphic adenoma; 2—mixed lipoepithelial tumors such as sialolipoma; 3—true adipose tumors like lipoma; and 4—fat-containing tumor-like salivary gland lesions in which diffuse lipomatosis falls [2].

Only a few cases of lipomatous pleomorphic adenomas and pleomorphic adenomas with extensive lipomatous metaplasia have been reported and well documented in the English literature [4–9].  Table 1 identifies these cases along with their attributes. Of the 8 previously reported cases, 3 were located in the parotid gland and 3 were located in the hard palate, similar to our case. Based on this information from a limited number of cases, there does not appear to be any significant sex predilection as 5 cases were in females and 3 occurred in males, as in our case.

The histogenesis of the LPA is not yet fully understood. The transformation of myoepithelial cells into fat cells (metaplasia) or the entrapment of adipose tissue during tumor expansion are both possible mechanisms [5, 6].

Pleomorphic adenomas, including the lipomatous variant, are generally treated by complete surgical excision. However, the tumor has a significant tendency to recur. PA recurrence can be related to multiple factors. Incomplete and intraoperative rupture of the tumor capsule, especially in cases of large parotid tumors involving the facial nerve, is an important factor [10] as is the size of the surgical margin [11]. Duration of the tumor prior to initial treatment is also a risk factor and it was found to be statistically significant by Wierzbicka et al. They found that the mean duration of the tumor in patients with recurrence was 66 months compared to a mean tumor duration of 28 months in patients without recurrence [10]. The main problem with recurrence is the increased risk of malignant transformation, which is estimated at 5-20% [12]. The World Health Organization (WHO) salivary gland tumor experts estimate that about 12% of carcinoma ex PA cases develop from recurrent PA tumors [13].

As mentioned previously, there is a concern that a long-standing pleomorphic adenoma can undergo malignant transformation. The transformation rate has been reported to range as low as 1.5% and as high as 13.8% [14]; the WHO reports a rate of 6.2% [13]. Furthermore, there appears to be a correlation between the malignant transformation rate and the clinical duration of the tumor. According to a review conducted by Khalesi, the incidence of malignant transformation increased from 1.6% for tumors present for less than 5 years to 9.6% for tumors present for over 15 years [14].

If malignant transformation of a PA to a carcinoma ex pleomorphic adenoma (CA ex PA) does occur, the treatment and prognosis radically change. Surgery followed by radiation treatment is considered as the standard of care for a patient with CA ex PA. Generally, patients with CA ex PA have a poor prognosis with common regional metastases and high mortality [15]. The WHO reports that local or distant metastasis occurs in as many as 70% of cases and the 5-year survival rate is 25-65% [13]. Therefore, early detection and proper complete surgical excision during the PA stage are of paramount importance.

Our case is also unique in that it is the only case of LPA initially diagnosed through punch biopsy to our knowledge. We advocate this technique as a quick and easy method to obtain the correct diagnosis so that appropriate treatment is not delayed.

Since the lipomatous pleomorphic adenoma is a fairly rare neoplasm, there were no reports of recurrence or malignant transformation in any of the reported LPA cases [4]. The patient in our report has also been free of any recurrence or malignant transformation to date.

## 4. Conclusion

Recognizing the varying presentations of salivary gland tumors is paramount for early diagnosis and treatment of the patient. Pleomorphic adenoma is the most common salivary gland tumor in the oral cavity, and it is of great importance to know its different clinical presentations. Performing a biopsy on any abnormality is the first step for treatment, especially since a lipomatous PA could mimic other more innocuous lesions such as lipoma and be missed on a CT scan due to the high fat content. Obtaining the definitive diagnosis was especially important in this case since long-standing pleomorphic adenomas have the potential to become malignant.

## Figures and Tables

**Table 1:** Currently reported cases of LPA and PA with significant adipose component.

Study	Patient demographics	Location of tumor	Microscopic diagnosis	Special features
Leroy et al. [8]	47 yo male	Parotid gland	Lipomatous PA	Adipose component of app 40%
Ide and Kusama [6]	35 yo female	Buccal mucosa	Myxolipomatous PA	10-year history, >95% adipose component
Haskell et al. [9]	33 yo female	Parotid gland	PA with extensive lipomatous metaplasia	Consisted of 60–70% lipocytic cells
Haskell et al. [9]	30 yo male	Upper lip	PA with extensive lipomatous metaplasia	Lipocytes comprised about 25% of tumor
Haskell et al. [9]	45 yo female	Hard palate	Myoepithelioma with extensive lipomatous metaplasia	Adipose component of 40% of the tumor, also showed osseous and cartilaginous areas
Kondo [5]	14 yo Japanese girl	Parotid gland	Lipomatous PA	Adipose component >95%, not picked up on CT due to fat content
Farah-Klibi et al. [7]	58 yo male	Hard palate	Lipomatous PA	Adipose component of app 30%
Musayev et al. [4]	32 yo female	Hard palate	Lipomatous PA	Fine-needle aspiration biopsy, excision showed >90% adipose tissue
Shah and Moustafa (2018)	24 yo Asian male	Hard palate	PA with significant adipose tissue component	Adipose component of app 50%
